# Association of cancer and outcomes of patients hospitalized for COVID-19 between 2020 and 2023

**DOI:** 10.12688/f1000research.150761.2

**Published:** 2025-04-07

**Authors:** Abdulai Tejan Jalloh, Laura Merson, Divya Nair, Shermarke Hassan, Ibrahim Franklyn Kamara, Innocent Nuwagira, Sia Morenike Tengbe, Yusuf Sheku Tejan, Mustapha Kabba, Sulaiman Lakoh, Donald S Grant, Robert J Samuels, Rugiatu Z Kamara, Robert F Terry

**Affiliations:** 1 Ministry of Health, Government of Sierra Leone, Freetown, Sierra Leone; 2 ISARIC, Pandemic Science Institute, University of Oxford, Oxford, England, UK; 3 International Union Against TB and Lung Disease, Paris, France; 4 Infectious Diseases Data Observatory, University of Oxford, Oxford, England, UK; 5 Centre for Tropical Medicine and Global Health, Nuffield Department of Medicine, University of Oxford, Oxford, England, UK; 6 World Health Organization, Freetown, Sierra Leone; 7 College of Medicine and Allied Health Sciences, University of Sierra Leone, Freetown, Western Area, Sierra Leone; 8 United States Centers for Disease Control and Prevention County Office, Freetown, Sierra Leone; 9 TDR, the Special Programme for Research and Training in Tropical Diseases, World Health Organization, Geneva, Switzerland

**Keywords:** COVID-19, cancer, comorbidities, mortality, hazard ratio, risk factor, ISARIC, SORT IT

## Abstract

**Background:**

The coronavirus disease 2019 (COVID-19) has caused substantial morbidity and mortality on a global scale. A strong correlation has been found between COVID-19 treatment outcomes and noncommunicable diseases such as cancers. However, there is limited information on the outcomes of cancer patients who were hospitalised for COVID-19.

**Methods:**

We conducted an analysis on data collected in a large prospective cohort study set-up by the International Severe Acute Respiratory and Emerging Infection Consortium (ISARIC). All patients with laboratory-confirmed or clinically-diagnosed SARS-CoV-2 infection were included. Cancer was defined as having a current solid organ or haematological malignancy. The following outcomes were assessed; 30-day in-hospital mortality, intensive care unit (ICU) admission, length of hospitalization and receipt of higher-level care.

**Results:**

Of the 560,547 hospitalised individuals who were analysed, 27,243 (4.9%) had cancer. Overall, cancer patients were older and had more comorbidities than non-cancer patients. Patients with cancer had higher 30-day in-hospital mortality than non-cancer patients (29.1.3% vs 18.0%) and longer hospital stays (median of 12 days vs 8 days). However, patients with cancer were admitted less often to intensive care units than non-cancer patients (12.6% vs 17.1%) and received less invasive mechanical ventilation than non-cancer patients (4.5% vs 7.6%). The hazard ratio of dying from cancer, adjusted for age, sex and country income level was 1.18 (95%CI: 1.15-1.2).

**Conclusions:**

This study's findings underscore the heightened vulnerability of hospitalized COVID-19 patients with cancer, revealing a higher mortality rate, longer hospital stays, and an unstructured pattern of care that reflects the complexity of managing severely ill patients during a public health crisis like the COVID-19 pandemic.

## Introduction

Early in the COVID-19 pandemic, data were collected to identify risk factors for poor outcomes that could inform a risk-based approach to health policy and patient management. Risk factors including age, sex, and several comorbidities were reported to be associated with an increased risk of death. ^1^^,^^2^ The most common comorbidities identified in hospitalised patients during the first wave of the COVID-19 pandemic were chronic cardiac or cardiovascular diseases, diabetes mellitus, hypertension, non-asthmatic chronic pulmonary disease, obesity, and chronic kidney disease. ^1^^,^^3^^–^^6^ Understanding which individuals are likely to have a poor prognosis could help inform vaccine prioritisation, shielding policies, or allocation of healthcare resources and patient management in future infectious disease outbreaks and pandemics.

Several studies have reported COVID-19 patients with cancer to be at higher risk of adverse outcomes compared with COVID-19 patients without cancer. ^7^^,^^8^ In a study from China, COVID-19 patients with cancer had higher observed increased rates of death, intensive care unit (ICU) admission, and need for invasive mechanical ventilation. ^9^ A study of COVID-19 patients in the United States of America reported that cancer patients were at higher risk of death and hospitalisation but were not found to have significantly different rates of ICU admission or ventilator use compared to non-cancer patients. ^10^ Data from the United States Centre for Disease Control showed that in 2020 and 2021 respectively, 2.0% and 2.4% of people who died of cancer had COVID-19 listed as the underlying cause of death. ^11^ There is a dearth of evidence on the outcomes of patients with cancer in middle- and low-income countries.

The studies referenced above and other national studies have shown that patients with cancer have worse outcomes than those without cancer when hospitalised due to COVID-19. ^12^^,^^13^ However, to our knowledge, no study has been conducted to evaluate the association between cancer and hospital outcomes among hospitalised COVID-19 patients using an international data set. This study, seeking to build on the collection of existing evidence, uses secondary COVID-19 patient data, collected in 54 countries via the Clinical Characterisation Protocol designed by the International Severe Acute Respiratory and Emerging Infections Consortium (ISARIC) and the World Health Organisation (WHO). ^14^^,^^15^ We investigated the association of cancer as a comorbidity with 30-day in-hospital mortality, ICU admission, length of hospitalization and receipt of higher-level care in COVID-19 patients with and without cancer.

## Methods

### Study design and setting

This was a prospective cohort study that utilised secondary data from the COVID-19 clinical database hosted by the Infectious Diseases Data Observatory (IDDO). The database contains individual patient data from more than 800,000 hospitalised patients in more than 1,200 institutions from 54 countries across 6 continents. The data were collected using the ISARIC-WHO case report form as a part of the ISARIC-WHO Clinical Characterisation Protocol. ^15^^,^^16^

### Study population

We included hospitalised patients of any age with clinically or laboratory-diagnosed SARS-CoV-2 infection. Patients were enrolled between 30 ^th^ January 2020 and 10 ^th^ January 2023. Patients with unknown cancer status were excluded. Patients admitted for complications due to COVID-19 were followed from the time of hospital admission to discharge or death.

### Study variables

We compared the differences in demographic characteristics, comorbidities, treatment with intensive interventions, length of hospitalisation, death (defined as 30-day in-hospital mortality), and hospital outcomes to characterise hospitalised COVID-19 patients with and without cancer.

Severe disease was defined as treatment with higher-level care, including one or more of the following events: admission to an ICU, treatment with invasive mechanical ventilation (IMV), non-invasive ventilation (NIV), high-flow nasal cannula (HFNC), inotropes and/or vasopressors. Length of hospital stay was censored at 100 days.

The presence of cancer was self-reported by patients or relatives and recorded as a binary variable classified as malignant neoplasm in the ISARIC-WHO case report form. Cancer was defined as having a current solid organ or haematological malignancy. Malignancies that had been declared ‘cured’ ≥5 years with no evidence of ongoing disease, non-melanoma skin cancer and benign growths or dysplasia were not included in this definition. Those with unknown cancer status were excluded.

### Data collection and validation

We used prospectively collected, international observational data on demographics, clinical features and outcomes of patients hospitalized with COVID-19 with or without cancer (coded as ‘malignant neoplasm’). Data were collected using the ISARIC-WHO Clinical Characterisation Protocol and contributed to a central repository at the University of Oxford, England. Participating sites used the ISARIC-WHO case report form to enter data onto a Research Electronic Data Capture (REDCap, https://www.project-redcap.org/ version 8.11.11, Vanderbilt University, Nashville, TN) database or used local databases before uploading to the central data repository. ^17^ Open Data Kit is a suitable open access alternative ( https://getodk.org). Centrally collated data were wrangled and mapped to the structure and controlled terminologies of the Study Data Tabulation Model ( https://www.cdisc.org/standards/foundational/sdtm, version 1.7, Clinical Data Interchange Standards Consortium, Austin, TX) using Trifacta ^®^ software version 9.7.1 ( http://trifacta.com). OpenRefine is a suitable open access alternative ( https://openrefine.org/) to using Trifacta ^®^. The data collection, aggregation, curation, and harmonisation process has been previously described. ^16^ Though more than 50% of the data were collected from low- and middle-income countries, most data on patients with cancer were collected from patients in higher income countries, per World Bank classification. Our statistical analysis plan was designed to explore differences between patient outcomes between these two economic regions as a proxy for the quality of the healthcare setting in a country.

### Analysis and statistical method

Continuous variables such as age and length of hospital stay were summarised as means with standard deviations or medians with interquartile ranges depending upon the distribution of data. Categorical variables (sex, presence of cancer, hospital exit outcomes, etc) were summarised as frequencies and percentages.

Categorical variables such as death and treatment with intensive interventions between patients with cancer and those without cancer were compared using the chi-square test. Continuous variables such as length of hospital stay were compared between the two groups using the unpaired t-test or Mann Whitney U test depending on the distribution of data. A Kaplan-Meier curve was plotted to show the cumulative incidence of mortality during hospitalization. To assess the independent effect of cancer on mortality in hospitalized COVID-19 patients, a survival analysis model was fitted to the data. The model was adjusted for the following confounders: age, sex, and country income-level with no explicit adjustment made for further co-morbidities. Unadjusted and adjusted hazard ratios with 95% confidence intervals were reported as measures of association. In addition, we undertook two further sensitivity analyses using different adjustment sets. Denominators on individual analyses differ due to availability of data on different variables across the dataset. A P-value of <0.05 was considered statistically significant.

Information on country income level was obtained from the World Bank ( https://datacatalog.worldbank.org/search/dataset/0038543).

All analyses were performed using R version 4.2.2 (IDE PBC, Boston, MA, USA), an open access software. (R: The R Project for Statistical Computing ( https://www.r-project.org/).

### Ethics considerations

Execution of the ISARIC-WHO Clinical Characterisation Protocol was approved by the WHO Ethics Review Committee (RPC571 and RPC572, 25 April 2013) and by local or national ethics committees for participating sites. Approvals (dates unknown) include the South Central—Oxford C Research Ethics Committee for England (Ref. 13/SC/0149), the Scotland A Research Ethics Committee (Ref. 20/SS/0028) for Scotland, and the Human Research Ethics Committee (Medical) at the University of the Witwatersrand in South Africa as part of a national surveillance programme (M160667), which collectively represent most of the data. Written patient consent for data to be collected and used in research was obtained or waived according to local norms determined by the responsible Ethics Committee. The data were collected using the ISARIC-WHO case COVID-19 report form, locally-tailored versions of the form, or independently designed forms. Arrangements surrounding the pooling, storage, curation and sharing of these data are covered by the IDDO Governance processes. ^18^

All data were deidentified and ensured of low risk for identification of individuals by a statistical disclosure process prior to sharing. Data were shared under a Data Access Agreement following approval from the IDDO Data Access Committee. ^19^ Execution of this secondary analysis was approved by the Union Ethics Advisory Group of the International Union against Tuberculosis and Lung Disease, Paris, France (EAG number 18/23, dated 8 ^th^ September 2023).

## Results

Among 841,640 individual records in the dataset, 560,547 (66.6%) met the criteria for analysis. Of those that did not, 73,327 (8.7%) did not have clinical or laboratory confirmation of SARS-CoV-2 infection; a further 3,879 (0.5%) were not admitted to hospitals between January 30 ^th^ 2020 and January 10 ^th^ 2023; and 203,887 (24.2%) did not have information on cancer status available.

### Demographics and comorbidities

Of the 560,547 individuals analysed, 27,243 (4.9%) had cancer. Furthermore, 219,922 (39.2%) individuals that met the criteria for analysis were hospitalised in high-income countries. There were differences in age, sex, country income level, and other comorbidities in the group of patients with cancer versus those without cancer. Those with cancer were older (84.4% versus 46.3% aged ≥60 years), were more likely to be male (58.1% versus 49.1%) and were more likely to come from a high-income country (90.6% versus 36.6%). Of the 10 comorbidities most common in the whole population, all except obesity were more prevalent in the group of patients with cancer ( Table 1).

**Table 1.** Demographic characteristics and comorbidities of COVID-19 patients with and without cancer hospitalised between 2020-2023 and enrolled to the ISARIC-WHO Clinical Characterisation Protocol.

	Cancer (N=27243)	Non-cancer (N=533304)
Age in years		
0-4	66 (0.2%)	9074 (1.7%)
5-14	153 (0.6%)	10811 (2.0%)
15-29	217 (0.8%)	39848 (7.5%)
30-44	824 (3.0%)	90335 (16.9%)
45-59	2981 (10.9%)	136110 (25.5%)
60 and above	23002 (84.4%)	247126 (46.3%)
Gender		
Male	15812 (58.1%)	261479 (49.1%)
Female	11395 (41.9%)	271435 (50.9%)
Countries, by income		
High income	24692 (90.6%)	195230 (36.6%)
Upper middle income	2497 (9.2%)	330196 (61.9%)
Lower middle income	29 (0.1%)	5684 (1.1%)
Low income	25 (0.1%)	2190 (0.4%)
Hypertension		
Yes	12037 (50.8%)	185555 (36.6%)
No	11681 (49.2%)	321205 (63.4%)
Chronic cardiac disease		
Yes	9018 (34.7%)	61224 (11.5%)
No	16965 (65.3%)	469010 (88.5%)
Smoking		
Yes	7674 (52.3%)	58420 (31.4%)
No	7005 (47.7%)	127878 (68.6%)
Diabetes		
Yes	7362 (28.0%)	124843 (23.9%)
No	18901 (72.0%)	397550 (76.1%)
Chronic pulmonary disease		
Yes	5168 (19.9%)	42312 (8.0%)
No	20812 (80.1%)	488232 (92.0%)
Chronic rheumatological disorder		
Yes	3727 (15.8%)	23328 (11.1%)
No	19867 (84.2%)	186487 (88.9%)
Chronic neurological disorder		
Yes	3179 (13.2%)	22432 (10.4%)
No	20831 (86.8%)	193715 (89.6%)
Dementia		
Yes	2954 (12.6%)	21656 (10.4%)
No	20569 (87.4%)	187540 (89.6%)
Asthma		
Yes	2654 (10.3%)	46029 (8.7%)
No	23144 (89.7%)	484495 (91.3%)
Obesity		
Yes	2411 (11.1%)	40334 (14.7%)
No	19390 (88.9%)	234157 (85.3%)

Note that denominators vary across variables dependant on data availability; Column percentages presented in the parentheses.

### Mortality, severity, and length of hospitalization

**Figure 1.**
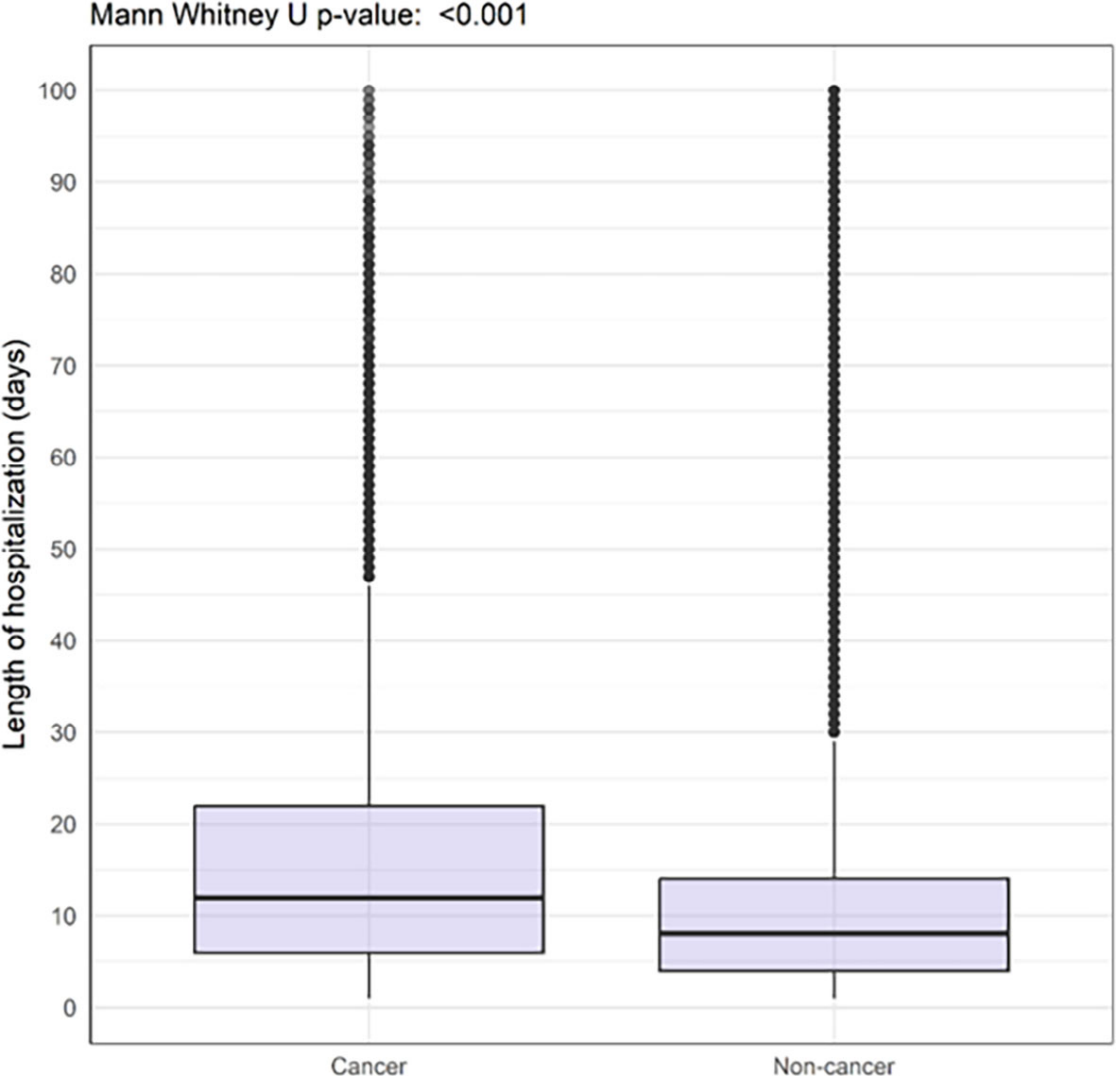
Boxplot showing length of hospitalisation among COVID-19 patients with and without cancer hospitalised between 2020-2023 and enrolled to the ISARIC-WHO Clinical Characterisation Protocol.

**Figure 2.**
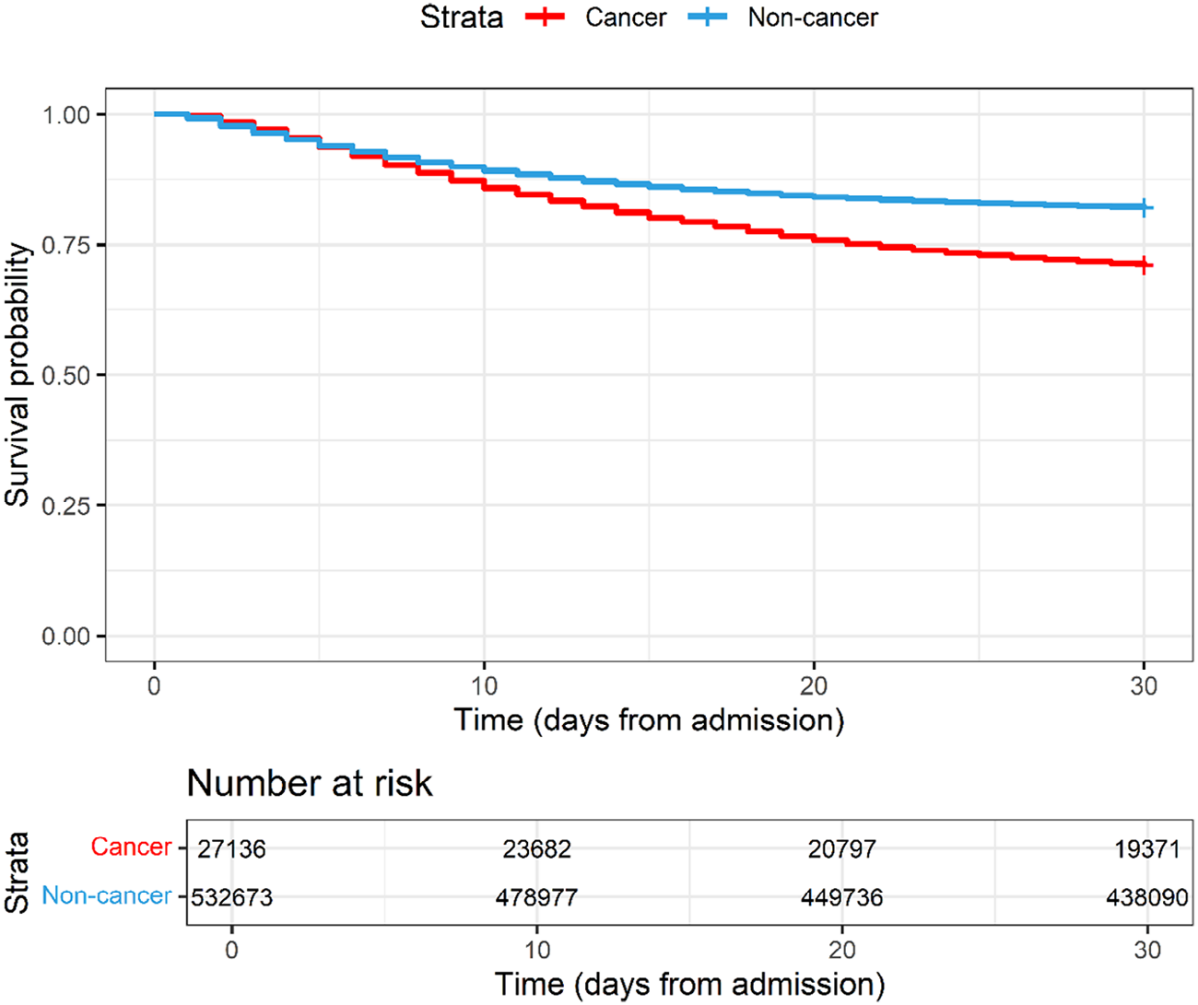
Kaplan-Meier plot of COVID-19 patients with and without cancer hospitalised between 2020-2023 and enrolled to the ISARIC-WHO Clinical Characterisation Protocol.

Patients with cancer had higher 30-day in-hospital mortality (29.1% vs 18.0%) and longer duration of hospitalization (median of 12 days (IQR 6.0-22.0) vs 8 days (IQR 4.0-14.0)) compared with those without cancer ( Table 2 and Figures 1 and 2).

**Table 2.** Mortality, hospital admission and high-level care in COVID-19 patients with and without cancer hospitalised between 2020-2023 and enrolled to the ISARIC-WHO Clinical Characterisation Protocol.

*All patients*	Cancer (N=27243)	Non-cancer (N=533304)
30-day in-hospital mortality		
Yes	7940 (29.1%)	95896 (18.0%)
No	19303 (70.9%)	437408 (82.0%)
Median duration of hospitalization (IQR) in days	12.0 (6.00, 22.0)	8.00 (4.00, 14.0)

*In the subset of patients who had data available on higher-level care*	Cancer (N=23994)	Non-cancer (N=271842)
Receipt of higher-level care		
Yes	6929 (28.9%)	81111 (29.8%)
No	17065 (71.1%)	190731 (70.2%)
Treated with high-flow nasal cannulas		
Yes	4201 (17.5%)	43680 (16.1%)
No	19793 (82.5%)	228162 (83.9%)
Admitted to ICU		
Yes	3023 (12.6%)	46372 (17.1%)
No	20971 (87.4%)	225470 (82.9%)
Treated with non-invasive ventilation		
Yes	2781 (11.6%)	31871 (11.7%)
No	21213 (88.4%)	239971 (88.3%)
Treated with invasive ventilation		
Yes	1070 (4.5%)	20545 (7.6%)
No	22924 (95.5%)	251297 (92.4%)
Treated with inotropes and/or vasopressors		
Yes	828 (3.5%)	12213 (4.5%)
No	23166 (96.5%)	259629 (95.5%)
Treated with extracorporeal membrane oxygenation		
Yes	30 (0.1%)	1232 (0.5%)
No	23964 (99.9%)	270610 (99.5%)

*Footnotes: IQR=interquartile range; ICU= intensive care unit;* Column percentages presented in the parentheses.

However, patients with cancer were reported to have received higher-level care slightly less often than those without cancer (28.9% vs 29.8%) including lower rates of ICU admission (12.6% vs 17.1%) and invasive mechanical ventilation (4.5% vs 7.6%). There were similar levels of treatment with high-flow nasal cannulas (17.5% vs 16.1%), extracorporeal membrane oxygenation (0.1% and 0.5%), non-invasive ventilation (11.6% vs 11.7%), and treatment with inotropes or vasopressors (3.5% vs 4.5%) across both groups ( Table 2).

The effect of cancer and other comorbidities on 30-day in-hospital mortality among COVID-19 patients is reported in Table 3. Hospitalised COVID-19 patients with cancer had a higher risk of 30-day in-hospital mortality compared to those without cancer. The hazard ratio of dying from cancer, adjusted for age, sex and country income level was 1.18 (1.15-1.2).

**Table 3.** Factors influencing 30-day in-hospital mortality among COVID-19 patients hospitalised between 2020-2023 and enrolled to the ISARIC-WHO Clinical Characterisation Protocol.

	Total (N=560547)	Deaths (N=103836)	Unadjusted hazard ratio (95% CI)	Adjusted hazard ratio * (95% CI)
Age				
60 years and above	270128	76514	2.01 (1.98-2.04)	2.43 (2.39-2.46)
0-59 years	290247	27309	ref	ref
Diabetes mellitus				
Yes	132205	34293	1.4 (1.38-1.42)	1.32 (1.31-1.34)
No	416451	67133	ref	ref
Chronic pulmonary disease				
Yes	47480	13571	1.31 (1.28-1.33)	1.30 (1.28-1.33)
No	509044	89157	ref	ref
Gender				
Male	277291	56727	1.11 (1.1-1.12)	1.19 (1.18-1.21)
Female	282830	47020	ref	ref
Cancer				
Yes	27243	7940	1.16 (1.13-1.18)	1.18 (1.15-1.2)
No	533304	95896	ref	ref
Chronic cardiac disease				
Yes	70242	20692	1.2 (1.19-1.22)	1.15 (1.13-1.17)
No	485975	81965	ref	ref
Obesity				
Yes	42745	8327	0.97 (0.95-0.99)	1.15 (1.13-1.18)
No	253547	48963	ref	ref
Hypertension				
Yes	197592	48449	1.37 (1.35-1.38)	1.13 (1.12-1.15)
No	332886	47568	ref	ref
Dementia				
Yes	24610	8212	1.51 (1.48-1.55)	1.08 (1.05-1.1)
No	208109	35207	ref	ref
Smoking				
Yes	66094	14328	1.04 (1.02-1.06)	1.06 (1.04-1.08)
No	134883	22727	ref	ref
Asthma				
Yes	48683	8790	0.93 (0.91-0.95)	1.04 (1.02-1.07)
No	507639	93839	ref	ref
Chronic neurological disorder				
Yes	25611	6384	1.13 (1.1-1.16)	1.02 (0.99-1.04)
No	214546	38379	ref	ref
Chronic rheumatological disorder				
Yes	27055	6398	1.13 (1.1-1.16)	0.96 (0.94-0.99)
No	206354	37244	ref	ref

* Adjustment made for age, sex and country income level.

Adjusted hazard ratios were higher for age and gender compared with those for cancer. Adjusted for sex and country income level, individuals aged ≥ 60 years had the highest hazard ratio 2.43 (2.39-2.46). Adjusted for age and country income level, male sex had a hazard ratio of 1.19 (1.18-1.21).

Among all comorbidities, only diabetes mellitus (HR: 1.32, 95%CI: 1.31-1.34) and chronic pulmonary disease (HR: 1.30, 95%CI: 1.28-1.33) were more strongly associated with an increased risk of death compared with cancer, after adjusting for age, sex and country income level. Two sensitivity analyses were conducted and the results are presented in Table 4 and Table 5. The findings of the sensitivity analyses indicate that the quantified hazards ratio for cancer remained unchanged when adjusted for different comorbidities (Tabel 4). In addition, the quantified association between any of the predictors and outcome remained relatively stable with some/minor differences in the estimated hazards ratio, apart from chronic neurological disorder ( Table 5). However, it has to be cautioned that such a multivariable model with all the predictors included is subject to large missingness.

**Table 4.** Hazards ratio of mortality among those with cancer, adjusted for comorbidities.

	Hazards ratio [95% confidence interval]
**Results presented in** Table 3	
Not adjusted for any variables (from Table 3)	1.16 (1.13-1.18)
Adjusted for age, sex, and income levels (from Table 3)	1.18 (1.15-1.20)
**Sensitivity analysis by adjusted for following comorbidities in addition to age, sex, and income levels:** hypertension, diabetes, COPD, obesity, chronic cardiac diseases, dementia, asthma, neurological disorder, rheumatological disorder	1.18 (1.14-1.21)

**Table 5.** Multivariable model with all the predictors listed in Table 3 included in the analysis (n=102,184 patients, 16,105 events, and 458,363 missing observations excluded from the multivariable analysis).

	Unadjusted hazards ratio (95% confidence interval) (from Table 3)	Adjusted hazards ratio (95% confidence interval)
Cancer (reference: no)	1.16 (1.13-1.18)	1.20 (1.16-1.26)
60 years and above (reference: 0-59y)	2.01 (1.98-2.04)	2.63 (2.50-2.77)
Diabetes mellitus (reference: no)	1.4 (1.38-1.42)	1.20 (1.17-1.24)
Chronic pulmonary disease (reference: no)	1.31 (1.28-1.33)	1.33 (1.28-1.38)
Male (reference: female)	1.11 (1.1-1.12)	1.24 (1.20-1.28)
Chronic cardiac disease (reference: no)	1.2 (1.19-1.22)	1.26 (1.21-1.30)
Obesity (reference: no)	0.97 (0.95-0.99)	1.10 (1.06-1.15)
Hypertension (reference: no)	1.37 (1.35-1.38)	1.10 (1.01-1.14)
Dementia (reference: no)	1.51 (1.48-1.55)	1.16 (1.10-1.22)
Smoking (reference: no)	1.04 (1.02-1.06)	1.04 (1.00-1.08)
Asthma (reference: no)	0.93 (0.91-0.95)	1.03 (0.98-1.08)
Chronic neurological disorder (reference: no)	1.13 (1.1-1.16)	0.95 (0.91-0.99)
Chronic rheumatological disorder (reference: no)	1.13 (1.1-1.16)	0.96 (0.93-1.00)

## Discussion

Our study findings underscore the heightened vulnerability of cancer patients hospitalized with COVID-19, revealing a higher mortality rate, longer hospital stays, and a nuanced pattern of care that reflects the complexity of managing severely ill patients during a public health crisis. These outcomes align with the existing literature on the association of cancer with COVID-19 prognosis and treatment approaches during the pandemic. ^13^^,^^20^ In keeping with our findings, other studies conducted in high-income countries have also documented that the proportion of COVID-19 patients with cancer and other comorbidities is higher in the elderly (>60 years) as compared to the general population. ^13^^,^^21^^,^^22^

A meta-analysis of 4 studies (4691 non-cancer patients, 154 cancer patients) that looked at mortality in cancer patients versus non-cancer patients reported a pooled odds ratio of death of 3.91 (95%CI: 2.70-5.67). ^12^ This is higher than reported in our study. This could be explained by the lack of adjustment for potential confounders in the meta-analysis. It is also unclear whether or not the patients in these studies were primarily admitted for COVID-19, for cancer, or for other reasons. When considering other significant risk factors for mortality, we observed that cancer ranked prominently. Cancer demonstrated a stronger association with mortality compared to all other comorbidities, except for diabetes mellitus and chronic pulmonary disease.

Despite the higher mortality risk, cancer patients in our study were slightly less likely to receive higher-level care compared to patients without cancer (28.9% vs 29.8%). Specifically, cancer patients were less frequently admitted to the ICU (12.6% vs. 17.1%) and had invasive mechanical ventilation less often (4.5% vs. 7.6%). These findings diverge from the expectation that higher-risk patients would necessitate more aggressive treatment. Though these event rates align with other studies of cancer patients, few comparators with non-cancer patients hospitalised for COVID-19 are in the literature. Marta et al. (2020) reported ICU admission rates of 39.1% in cancer patients with COVID-19 and use of invasive mechanical ventilation in 84.4%. ^23^ Elgohary et al.’s (2021) systematic review and meta-analysis of cancer patients with COVID -19 reported an ICU admission rate of 14.5% (95% CI: 8.5-20.4) and a mechanical ventilation rate of 11.7% (95% CI: 5.5-18). ^12^ When comparing cancer patients with non-cancer patients, Abuhelwa et al. (2022) found cancer patients hospitalized for COVID-19 had similar rates of invasive mechanical ventilation compared to those without cancer (10.14% vs 9.36.%). ^13^

We found differences in mean hospital stay between patients with cancer and those without cancer. The longer hospital stay might be related to cancer patients having several other comorbidities and/or the cancer-related management. However, we cannot explain why they stayed longer in hospital but received less high-level care compared to COVID-19 patients without cancer. Abuhelwa’s 2022 nation-wide study reported no difference in hospital stays between these patient groups (8.07 vs 7.46 days). The difference between these findings and ours may reflect differences in admission policy or availability of hospital beds. The lower mortality rates in Abuhelwa’s study as compared to our findings may indicate less severe disease, and therefore a population requiring less in-hospital care.

### Strengths and limitations

One key strength of our study was the use of a large sample size, orders of magnitude larger than most previous studies. Therefore, our estimates should be more generalisable and should have a higher power to demonstrate significant associations than previously published studies. We adhered to the STROBE (Strengthening the Reporting of Observational Studies in Epidemiology) guidelines for reporting study findings.

This analysis has several limitations. Though recruitment of patients included in the database used for this analysis targeted those admitted for complications due to COVID-19, the reason for other admissions is not recorded in the database and therefore we cannot verify other reasons for admission but analysed the hospital outcomes of patient with cancer and COVID -19. Any inclusion of patients admitted for other reasons may have had an impact on the subsequent treatment pathway. This study encompassed all patients with current cancer, but we couldn't distinguish between various cancer types as detailed information on each patients’ cancer diagnosis, staging and treatment modalities were not available. However, other studies have highlighted lung cancer and haematological cancers as being those most closely linked to mortality in COVID-19 patients. ^13^^,^^24^^,^^25^

Differences in reporting of the type and details of cancer diagnosis across the available literature make it challenging to make comparisons. Studies that analysed data through the use of electronic health records may have included patients in remission.

Our study includes patients from January 30, 2020, to January 10, 2023. During this period, COVID-19 underwent significant changes in genomics, treatment, and epidemiology, with vaccines introduced at varying times across countries. However, our dataset lacks genotyping and reliable vaccination information, which are crucial for analysing temporal changes accurately. Without data on these key factors, especially vaccination status, we cannot provide a robust analysis of changes over time. The impact of evolving vaccination rates on outcomes is likely substantial but impossible to calculate with our current data.

We acknowledge this limitation, and it has informed changes to ISARIC's case report forms for future outbreaks to address these data gaps. The majority of data on patients with cancer (90.6%) were collected from patients in high income countries. So, no inferences could be drawn from patient outcomes linked with World Bank income classifications.

## Conclusions

Our study found that patients with cancer were older with more comorbidities. They had an increased risk of mortality with longer duration of hospital stay as compared to non-cancer patients but received less high-level care including ICU admission and invasive mechanical ventilation. This highlights the importance of collecting accurate data in emerging infections to identify at-risk groups, facilitating appropriate resource allocation and patient management and informing policy decisions aimed at resource allocation during health emergencies. The availability and collection of data on our platforms were predominantly from high-income countries. To prepare for a future pandemic, data availability and coverage must be more universal. More must also be done to support data collection and the capacity to analyse those data within low- and middle-income countries for appropriate evidence generation and proper patient care.

## Author contributions

Conceptualization and methodology, ATJ, LM, DN, SH, YST, RFT; formal analysis and visualisation, DN, SH; supervision, project administration and funding acquisition, RFT, LM; writing—original draft preparation, ATJ, LM, SH, RFT; writing—review and editing, ATJ, LM, DN, SH, IFK, IN, SMT, YST, MK, SL, DSG, RJS, RK, RFT. All authors have read and agreed to the last version of the manuscript.

## Open access statement

In accordance with WHO’s open-access publication policy for all work funded by WHO or authored/co-authored by WHO staff members, WHO retains the copyright of this publication through a Creative Commons Attribution IGO license ( http://creativecommons.org/licenses/by/3.0/igo/legalcode) which permits unrestricted use, distribution and reproduction in any medium provided the original work is properly cited.

## Data Availability

The data that underpin this analysis are available via a governed data access mechanism following review of a data access committee. Data can be requested via the IDDO COVID-19 Data Sharing Platform ( http://www.iddo.org/covid-19 ). The Data Access Application, Terms of Access and details of the Data Access Committee are available on the website. Briefly, the requirements for access are a request from a qualified researcher working with a legal entity who have a health and/or research remit; a scientifically valid reason for data access which adheres to appropriate ethical principles. The full terms are at: https://www.iddo.org/document/covid-19-data-access-guidelines . These data are a part of https://doi.org/10.48688/cpwp-ft84.
