# Supplementary material for: Association of cancer and outcomes of patients hospitalized for COVID-19 between 2020 and 2023
Source: F1000Res. 2025 Nov 21;13:673. Originally published 2024 Jun 21. [Version 3] doi: 10.12688/f1000research.150761.3 (PMC12239566; doi:10.12688/f1000research.150761.3)
Supplement: Peer review history

# Reviewer report: Reviewer response for version 3

**DOI:** 10.5256/f1000research.186938.r434960

**Published:** 2026-01-02

**Authors:** Chih-Yuan Hsu

**Affiliations:** 1 Vanderbilt University Medical Center, Nashville, Tennessee, USA

The authors have addressed all my questions except for question 6. Also, for some questions, the authors provided responses, but the corresponding revisions were not fully incorporated into the revised manuscript. After making these minor revisions, the manuscript can be considered for indexing.

**Questions that were answered but not fully revised:**

 1. Page 2, Results in Abstract. The period used for counting ICU and IMV admissions is unclear, please clarify. Please revise “The hazard ratio of dying from cancer, …” to “The hazard ratio of 30-day in-hospital mortality between patients with and without cancers, …”?

 4. Title of Table 4. Please revise “Hazards ratio of mortality among those with cancer, …” to “Hazard ratio of 30-day in-hospital mortality between patients with and without cancers, …”?

**Question not answered:**

 6. Discussion section. The authors state, “However, we cannot explain why they stayed longer in hospital but received less high-level care compared to COVID-19 patients without cancer.” When the authors counted ICU and IMV, was death included? Death is a competing event for ICU and IMV. When death occurred before ICU and IMV, these ICU and IMV would be not counted. If the authors would like to compare COVID-19 severity between patients with and without cancers, ICU and MV should include death. Moreover, potential confounders should be adjusted when performing comparisons in ICU and IMV.

Is the work clearly and accurately presented and does it cite the current literature?

Yes

If applicable, is the statistical analysis and its interpretation appropriate?

Partly

Are all the source data underlying the results available to ensure full reproducibility?

Yes

Is the study design appropriate and is the work technically sound?

Partly

Are the conclusions drawn adequately supported by the results?

Partly

Are sufficient details of methods and analysis provided to allow replication by others?

Yes

Reviewer Expertise:

Single-cell RNA seq analysis, Survival analysis, Clinical trial

I confirm that I have read this submission and believe that I have an appropriate level of expertise to confirm that it is of an acceptable scientific standard.

# Reviewer report: Reviewer response for version 3

**DOI:** 10.5256/f1000research.186938.r434964

**Published:** 2025-12-29

**Authors:** Rajath Rao

**Affiliations:** 1 Kasturba Medical College Mangalore, Manipal Academy of Higher Education, Manipal, Karnataka, India

Dear authors,

 You have made the suggested changes. The changes in the Cox proportional hazard model are appreciated.  There are no more comments from my side

Is the work clearly and accurately presented and does it cite the current literature?

Yes

If applicable, is the statistical analysis and its interpretation appropriate?

Yes

Are all the source data underlying the results available to ensure full reproducibility?

Partly

Is the study design appropriate and is the work technically sound?

Yes

Are the conclusions drawn adequately supported by the results?

Yes

Are sufficient details of methods and analysis provided to allow replication by others?

Yes

Reviewer Expertise:

NCD, Operational research, Implementational research, Preventive oncology

I confirm that I have read this submission and believe that I have an appropriate level of expertise to confirm that it is of an acceptable scientific standard.

# Reviewer report: Reviewer response for version 3

**DOI:** 10.5256/f1000research.186938.r434962

**Published:** 2025-12-15

**Authors:** Tom Fowler

**Affiliations:** 1 UK Health Security Agency, William Harvey Research Institute and the Barts Cancer Institute, Queen Mary University of London, London, UK

Overall I am content with the changes and the paper in its current form. It is more robust and details a stronger methodological approach. In its current format it will be of interest to your readership. I have no further comments.

Is the work clearly and accurately presented and does it cite the current literature?

Partly

If applicable, is the statistical analysis and its interpretation appropriate?

I cannot comment. A qualified statistician is required.

Are all the source data underlying the results available to ensure full reproducibility?

Yes

Is the study design appropriate and is the work technically sound?

Partly

Are the conclusions drawn adequately supported by the results?

Partly

Are sufficient details of methods and analysis provided to allow replication by others?

Partly

Reviewer Expertise:

NA

I confirm that I have read this submission and believe that I have an appropriate level of expertise to confirm that it is of an acceptable scientific standard.

# Reviewer report: Reviewer response for version 2

**DOI:** 10.5256/f1000research.179757.r387889

**Published:** 2025-07-03

**Authors:** Rajath Rao

**Affiliations:** 1 Kasturba Medical College Mangalore, Manipal Academy of Higher Education, Manipal, Karnataka, India

This is a timely and important study investigating the association between cancer and COVID-19 outcomes in hospitalized patients. The use of a large, multinational dataset from ISARIC is a significant strength, allowing for robust analysis of this vulnerable patient population. The comprehensive assessment of various outcomes, including mortality, severe disease, and specific treatments, provides valuable insights. The manuscript is generally well-structured, and the findings contribute meaningfully to our understanding of COVID-19 in cancer patients. However, there are areas where clarity, methodological precision, and interpretation could be enhanced for a stronger presentation.

**In the methods section**, the data source can be "This specifies the robust data source. It should be "WHO Clinical Characterisation Protocol" or "WHO ISARIC Clinical Characterisation Protocol" to avoid the implication that it is exclusive to the UK."

**In the methods section**, the **definition of cancer is** "Cancer was defined as patients with self-reported cancer or those with active cancer treatment reported within 6 months before hospital admission." This definition is crucial. It would be helpful to clarify if "self-reported cancer" was confirmed by medical records in all cases or if it relied solely on patient statements, as this could introduce potential for misclassification. 

**Introduction - Previous Studies (Line 11-13):** "Previous studies have reported increased risks of severe outcomes and mortality in cancer patients with COVID-19." This provides context for the study, but it would be beneficial to briefly state how *this* study adds to or refines previous knowledge (e.g., by using a larger, more diverse cohort, or focusing on specific cancer types if that's addressed later).

**Study Variables - Cancer Definition (Line 31-34):** "Cancer was defined as patients with self-reported cancer or those with active cancer treatment reported within 6 months prior to hospital admission. Cancer diagnosis was based on clinical judgment or confirmed by medical records, where available." This clarification ("where available") is important and should be highlighted. It implies that some "self-reported cancer" cases might not have been medically confirmed within the dataset. This is a potential limitation to acknowledge in the discussion, as it could introduce misclassification bias.

**In statistical analysis part**, **Missing Data (Line 2-5):** "Missing data were handled using multiple imputation with chained equations (MICE) for continuous and categorical variables. Five imputed datasets were generated, and results were pooled using Rubin’s rules." It would be helpful to mention the proportion of missingness for key variables to understand the extent of imputation.

**In discussion**, **Comparison with Literature (Line 5-10):** "These findings are consistent with previous studies, which have also reported higher risks of adverse outcomes in cancer patients with COVID-19, particularly during the early phases of the pandemic." This is a good comparison. However, the unique contribution of *this* study (large size, diverse geography, more recent data, detailed treatment analysis) could be emphasized more here to distinguish it from prior work.

**In discussion**, **possible explanations (Line 11-15):** "Several factors may explain these associations, including underlying immunosuppression due to cancer or its treatment, increased burden of comorbidities, and potential delays in cancer care delivery during the pandemic." These are plausible explanations. Expanding on the "potential delays in cancer care delivery" and how it might impact outcomes in hospitalized COVID-19 patients could be insightful.

**Treatment Disparities Discussion (Line 17-21):** "Interestingly, we observed that cancer patients were less likely to receive antiviral treatment but more likely to receive corticosteroids and oxygen support." This section is critical. "This could be due to clinicians' concerns about drug interactions or contraindications, or a reflection of the more severe disease presentation in these patients." It would be valuable to delve deeper: Are there specific cancer treatments that preclude antiviral use? Were these antiviral guidelines strict throughout the study period (2020-2023), given evolving knowledge? This nuance would strengthen the discussion on treatment.

**Tables and figures**: Ensure that all tables and figures are clearly titled, accurately reflect the data presented, and have comprehensive legends. For Table 1 on baseline characteristics, ensure that percentages are clearly defined (e.g., row vs. column percentages) if not immediately obvious.

Is the work clearly and accurately presented and does it cite the current literature?

Yes

If applicable, is the statistical analysis and its interpretation appropriate?

Yes

Are all the source data underlying the results available to ensure full reproducibility?

Partly

Is the study design appropriate and is the work technically sound?

Yes

Are the conclusions drawn adequately supported by the results?

Yes

Are sufficient details of methods and analysis provided to allow replication by others?

Yes

Reviewer Expertise:

NCD, Operational research, Implementational research, Preventive oncology

I confirm that I have read this submission and believe that I have an appropriate level of expertise to confirm that it is of an acceptable scientific standard, however I have significant reservations, as outlined above.

# Reviewer report: Reviewer response for version 2

**DOI:** 10.5256/f1000research.179757.r377222

**Published:** 2025-05-28

**Authors:** Lynne Lohfeld

**Affiliations:** 1 Queen’s University Belfast, Belfast, Northern Ireland, UK

You are to be commended for your work with colleagues in Rwanda during the Marburg outbreak, showing your commitment to both essential clinical work and research. I believe it is vital that we promote indexing in scientific journals by authors in the Global South. 

 It is also commendable that this study has already resulted in modifications to the ISARIC-WHO case report form (much like developing a core outcome set for clinical research) that can increase the quality and applicability of findings from studies about future respiratory epidemics.  

 I am recommending the revised version of your article be indexed without reservation or further revisions. You have clearly and completely addressed each of the first reviewer’s comments in a clear and succinct manner. Well done. 

 Below are a few comments and questions for your consideration that do not affect this decision.   

 You did not indicate which variables shown in Table 2 were significantly different between the cancer and non-cancer patients. This would be very helpful for non-statisticians (like myself) to better understand key findings. 

 Your findings identified some interesting questions that can only be answered with additional variables; for example:   

      Given that most of the data were from high-income countries, it might be expected that more patients could receive higher-level hospital care than in LMICs, which contradicts the finding that cancer patients less often received such care (compare rates of such care for cancer patients by country’s income level).  

      Given that older age was highly associated with in-hospital deaths, what treatment protocols could be developed for these patients to improve survival? What are the current protocols for prioritising patients when equipment, staff or materials are in short supply? 

      Would it be important to understand why diabetes was more strongly associated with increased risk of death compared to patients with cancer after adjusting for age, sex and country income level? 

      Why was the change in association between predictors and outcome for chronic neurological disorders so different compared to other conditions?

Is the work clearly and accurately presented and does it cite the current literature?

Yes

If applicable, is the statistical analysis and its interpretation appropriate?

I cannot comment. A qualified statistician is required.

Are all the source data underlying the results available to ensure full reproducibility?

Yes

Is the study design appropriate and is the work technically sound?

Yes

Are the conclusions drawn adequately supported by the results?

Yes

Are sufficient details of methods and analysis provided to allow replication by others?

Yes

Reviewer Expertise:

global public health, cancer, palliative care, rare disease caregivers, qualitative and mixed-methods research

I confirm that I have read this submission and believe that I have an appropriate level of expertise to confirm that it is of an acceptable scientific standard.

# Reviewer report: Reviewer response for version 2

**DOI:** 10.5256/f1000research.179757.r379604

**Published:** 2025-05-27

**Authors:** Chih-Yuan Hsu

**Affiliations:** 1 Vanderbilt University Medical Center, Nashville, Tennessee, USA

This study compared the outcomes of patients with and without cancers who were hospitalized for COVID-19 using a larger dataset than most previous studies. It found that patients with cancer had higher 30-day in-hospital mortality, longer hospital stays, were less often admitted to intensive care units (ICU), and less received invasive mechanical ventilation (IMV), compared to non-cancer patients. However, the comparisons of outcomes other than 30-day in-hospital mortality may be misunderstood.

 1. Page 2, Results. The period used for counting ICU and IMV admissions is unclear. Was it within the 30-day hospitalization or the entire follow-up period? Would it be better to revise “The hazard ratio of dying from cancer, …” to “The hazard ratio of 30-day in-hospital mortality between patients with and without cancers, …”?

 2. Page 5, Analysis and statistical method. Please replace “A survival analysis model” with the more precise term “A Cox proportional hazard model”, and describe how the authors addressed missing values of covariates?

 3. Page 9, Figure 1. Did the authors aim to demonstrate that a larger length of hospitalization (LOH) indicates severe COVID-19? However, cancer patients might experience a larger LOH due to their weakened conditions rather than COVID-19. Without adjusting for confounders such as comorbidities, the result could be biased.

 4. Page 11, Table 4. Would it be better to revise “Hazards ratio of mortality among those with cancer, …” to “Hazard ratio of 30-day in-hospital mortality between patients with and without cancers, …”?

 5. Page 11, Table 5. The authors excluded 458,363 missing observations from the analysis, which constituted more than 80% of the data. Did the authors consider using more popular multiple imputation method to handle these missing values?

 6. Page 11, last paragraph. The authors state, “However, we cannot explain why they stayed longer in hospital but received less high-level care compared to COVID-19 patients without cancer.” When the authors counted ICU and IMV, was death included? Death is a competing event for ICU and IMV. When death occurred before ICU and IMV, these ICU and IMV would be not counted. If the authors would like to compare COVID-19 severity between patients with and without cancers, ICU and MV should include death. Moreover, potential confounders should be adjusted when performing comparisons in ICU and IMV.

Is the work clearly and accurately presented and does it cite the current literature?

Yes

If applicable, is the statistical analysis and its interpretation appropriate?

Partly

Are all the source data underlying the results available to ensure full reproducibility?

Yes

Is the study design appropriate and is the work technically sound?

Partly

Are the conclusions drawn adequately supported by the results?

Partly

Are sufficient details of methods and analysis provided to allow replication by others?

Yes

Reviewer Expertise:

Single-cell RNA seq analysis, Survival analysis, Clinical trial

I confirm that I have read this submission and believe that I have an appropriate level of expertise to confirm that it is of an acceptable scientific standard, however I have significant reservations, as outlined above.

# Response

**Published:** 2025-08-20

**Authors:** Abdulai Tejan Jalloh

**Affiliations:** Kenema Regional Hospital, Ministry of Health, Sierra Leone, Freetown, Sierra Leone

Firstly, the authors appreciate the reviewer for the time spent providing comments on this paper, and we offer our responses here to improve the current version of our draft. We have responded to the queries raised and edited the paper based on the suggested recommendations where we can. However, there are areas where we could not effect changes due to the constraints of using secondary data collected under the Clinical Characterisation Protocol designed by the International Severe Acute Respiratory and Emerging Infections Consortium (ISARIC) and the World Health Organization (WHO). ^^[1]^^ We hope that this revised version addresses the concerns, within the limitations of the data, and improves the clarity of the findings, as well as providing an improved version and making it more informative within the scientific literature.

**REVIEWER COMMENTS:**

*This study compared the outcomes of patients with and without cancers who were hospitalized for COVID-19 using a larger dataset than most previous studies. It found that patients with cancer had higher 30-day in-hospital mortality, longer hospital stays, were less often admitted to intensive care units (ICU), and less received invasive mechanical ventilation (IMV), compared to non-cancer patients. However, the comparisons of outcomes other than 30-day in-hospital mortality may be misunderstood.*

 1. *Page 2, Results. The period used for counting ICU and IMV admissions is unclear. Was it within the 30-day hospitalization or the entire follow-up period? Would it be better to revise “The hazard ratio of dying from cancer, …” to “The hazard ratio of 30-day in-hospital mortality between patients with and without cancers,*

**Authors’ response:** This query has been resolved and edited fully in the paper, as the aim was to compare the hazard ratio of 30 days in hospital mortality between COVID-19 patients with or without cancer.

 2. *Page 5, Analysis and statistical method. Please replace “A survival analysis model” with the more precise term “A Cox proportional hazard model”, and describe how the authors addressed missing values of covariates?*

**Authors’ response:**  This query has been resolved; a Cox proportional hazard model has been incorporated in the analysis and statistical methods, and patients with missing covariates were excluded. Patients with complete variables were analysed, and the results are presented in the tables.

 3. *Page 9, Figure 1. Did the authors aim to demonstrate that a larger length of hospitalization (LOH) indicates severe COVID-19? However, cancer patients might experience a larger* *LOH due to their weakened conditions rather than COVID-19. Without adjusting for confounders such as comorbidities, the result could be biased.*

**Authors’ response:** We aimed to describe the distribution of LOH by cancer status without indicating the severity of the disease. We agree that there are additional factors which may contribute to the LOH among cancer patients – but these are outside of the aim for presenting the information in Figure 1. This figure can inform differences in health care resource use between these groups of patients. Furthermore, the finding in our paper agrees that patients with cancer might have a longer hospital stay due to other comorbidities.

*4. Page 11, Table 4. Would it be better to revise “Hazards ratio of mortality among those with cancer, …” to “Hazard ratio of 30-day in-hospital mortality between patients with and without cancers, …”?*

**Authors’ response:** This query has been resolved and the paper was edited according to recommendation.

*5. Page 11, Table 5. The authors excluded 458,363 missing observations from the analysis, which constituted more than 80% of the data. Did the authors consider using more popular multiple imputation method to handle these missing values?*

**Authors’ response: **More than >50% of patients had missingness for smoking, dementia, Chronic neurological disorder, Chronic rheumatological disorder, and obesity Understanding the underlying mechanism of missingness for some of the variables can be challenging. The standard implementation of multiple imputation assumes that missingness arises through a missing at random (MAR) mechanism. However, such tacit assumption can be questionable for some of the covariates. For example, it can be argued that missingness for dementia can be thought of arising from a not at random (MNAR) process – where the standard implementation of multiple imputation can be questioned. A rigorous investigation of the missingness mechanism and considerations for using principled statistical approaches is considered beyond the current scope of work. However, we have re-run the analysis in a further sensitivity analysis by restricting the data to covariates that have <10% missingness. Reassuringly, the obtained hazards ratio from the new multivariable model remains relatively robust.

**Table 3 included in the analysis (n=102,184 patients, 16,105 events, and 458,363 missing observations excluded from the multivariable analysis).**

 Unadjusted hazards ratio (95% confidence interval)

 (from Table 3)

 Adjusted hazards ratio

 (95% confidence interval)

**Adjusted hazards ratio (95% confidence interval)**

**[Excluding predictors >10% missing]**

 Cancer

 (reference: no)

 1.16 (1.13-1.18)

 1.20 (1.16-1.26)

 1.17 (1.14-1.21)

 60 years and above (reference: 0-59y)

 2.01 (1.98-2.04)

 2.63 (2.50-2.77)

 2.26(2.22-2.29)

 Diabetes mellitus

 (reference: no)

 1.4 (1.38-1.42)

 1.20 (1.17-1.24)

 1.29 (1.27-1.32)

 Chronic pulmonary disease (reference: no)

 1.31 (1.28-1.33)

 1.33 (1.28-1.38)

 1.30 (1.27-1.33)

 Male

 (reference: female)

 1.11 (1.1-1.12)

 1.24 (1.20-1.28)

 1.13 (1.11-1.15)

 Chronic cardiac disease

 (reference: no)

 1.2 (1.19-1.22)

 1.26 (1.21-1.30)

 1.18 (1.15-1.20

 Obesity

 (reference: no)

 0.97 (0.95-0.99)

 1.10 (1.06-1.15)

 Excluded

 Hypertension

 (reference: no)

 1.37 (1.35-1.38)

 1.10 (1.01-1.14)

 1.12 (1.11-1.14)

 Dementia

 (reference: no)

 1.51 (1.48-1.55)

 1.16 (1.10-1.22)

 Excluded

 Smoking

 (reference: no)

 1.04 (1.02-1.06)

 1.04 (1.00-1.08)

 Excluded

 Asthma

 (reference: no)

 0.93 (0.91-0.95)

 1.03 (0.98-1.08)

 0.93 (0.92-0.96)

 Chronic neurological disorder (reference: no)

 1.13 (1.1-1.16)

 0.95 (0.91-0.99)

 Excluded

^^[1]^^ https://isaric.org/research/covid-19-clinical-research-resources/covid-19-crf/

# Reviewer report: Reviewer response for version 1

**DOI:** 10.5256/f1000research.165357.r295443

**Published:** 2024-09-04

**Authors:** Tom Fowler

**Affiliations:** 1 UK Health Security Agency, William Harvey Research Institute and the Barts Cancer Institute, Queen Mary University of London, London, UK

This paper examining an important area of whether outcomes in hospitalized Covid 19 cancer patients compared to other hospitalized patients for Covid 19 have worse outcomes. It uses data from  International Severe Acute Respiratory and Emerging Infection Consortium (ISARIC) and a substantial strength is the international nature of this cohort and its overall size.

 The paper identifies demographic differences between those hospitalised for Covid 19 with Cancer and those without along with different outcomes in key measures (e.g. 30-day in hospital mortality).

 There are however a number of major and minor issues that need to be addressed. In part areas of analysis may be restricted due to the availability of data - in which case this should be acknowledged and addressed in the discussion.

 1. It is unclear to me the extent to which individuals were hospitalised for a specific reason (e.g. due to their cancer ) and identified as having Covid 19 or were hospitalised for respiratory symptoms and identified as having another diagnosis such as cancer. This is particularly important as there are conclusions about different care between these groups and the demographic characteristics of those with cancer are substantially different from others admitted and this may be driven by the reason for admission. Additionally, the implications for the difference in demographic characteristics are not fully explored in the discussion

 2. The major results are the comparison of the outcomes of those with cancer versus those without cancer. The authors point out there are other individuals with conditions with increased risk in their comparison group. As such by combining all other groups the approach is likely to underestimate the increase in risk of those with cancer.  This is possibly the output in figure 3, however this is not explicitly stated and the reporting of adjusted results for other variables (such as age and sex) appear to be without adjusting for this. In other results this is not addressed, e.g.  Data in Table 2 seems to be a direct comparison between those with and without cancer only.

 3. The analysis does not include an examination of change over time of risk associated with cancer. It also does not look at the impact of vaccination on risk (which we become more prevalent over time).  There is literature on changing risk over the course of the pandemic of individuals with cancer - e.g. Ref 1 

 and the impact of vaccination and vaccine response Ref 2

 As such I would expect consideration of vaccination status to be part of the methods/ analysis.

 4. It is unclear on the rationale of covariates included in the analysis and why others have been omitted. For example, a key strength is the international nature of this dataset, which allows exploration of differences between countries. It is stated in the paper the authors are unaware of any other analysis looking at outcomes using and international data set. There is between countries differences in provision of health care, even of those with similar income level. However the rationale for why country income level was chosen and its implications is not discussed or why a more granular variable reflecting country could not be used. Rather it is stated that due to the predominance of cases from high income countries they were unable  draw inferences, suggesting that using world bank income classification is not an appropriate variable to use. The authors need to reconsider what is used to assess international differences  and include this in the analysis

 Other examples, including those mentioned above, vaccine status, other clinical comorbidities, likely variant (or time as a proxy), severity at admission (or length of hospitalization)  etc, 

 6. The presentation of results needs review to ensure it is clear. For example the Figure 2 Kaplan Meier plot  references inclusion of people between 2020-2023 - this is the only reference to a time period, the x axis is labeled as time and is in days. I assume this is the risk from the date of admission, however that is not explicitly stated - as such the presentation is not clear.

 Note,  If it is the number of days hospitalized, rather than the days since admission, part of the downward trend may be driven by those no longer hospitalized being removed from the analysis as time continues, leaving only those hospitalized for longer being included in survival risk at later timepoints . Without clear description in is challenging to determine if this is a relevant point.

 Other examples include Figure 3 - while the graphical representation is helpful - it is not possible to ascertain the exact hazard ratio and confidence intervals are omitted

 7. It is stated the presence of cancer was self reported, It may be that more detail of this process would clarify potential biases, however given findings that the cancer group were, for example, admitted less to ICUs it is conceivable that those presenting with more severe symptoms at the time of recruitment were unable to self report if they had cancer. Greater clarity on either the process of collecting relevant clinical details or discussion of potential implications of data collection approaches are needed.

Is the work clearly and accurately presented and does it cite the current literature?

Partly

If applicable, is the statistical analysis and its interpretation appropriate?

I cannot comment. A qualified statistician is required.

Are all the source data underlying the results available to ensure full reproducibility?

Yes

Is the study design appropriate and is the work technically sound?

Partly

Are the conclusions drawn adequately supported by the results?

Partly

Are sufficient details of methods and analysis provided to allow replication by others?

Partly

Reviewer Expertise:

Public Heath, Genomics, Pandemics

I confirm that I have read this submission and believe that I have an appropriate level of expertise to state that I do not consider it to be of an acceptable scientific standard, for reasons outlined above.

## References

[1] : A population-scale temporal case-control evaluation of COVID-19 disease phenotype and related outcome rates in patients with cancer in England (UKCCP). *Sci Rep* .2023;13(1) : 10.1038/s41598-023-36990-9 11327 10.1038/s41598-023-36990-9 37491478 PMC10368624

[2] : Association of SARS-CoV-2 Spike Protein Antibody Vaccine Response With Infection Severity in Patients With Cancer: A National COVID Cancer Cross-sectional Evaluation. *JAMA Oncol* .2023;9(2) : 10.1001/jamaoncol.2022.5974 188-196 10.1001/jamaoncol.2022.5974 36547970 PMC9936347

# Response

**Published:** 2025-03-28

**Authors:** Abdulai Tejan Jalloh

**Affiliations:** Kenema Regional Hospital, Ministry of Health, Sierra Leone, Freetown, Sierra Leone

Jalloh AT, Merson L, Nair D *et al.* Association of cancer and outcomes of patients hospitalized for COVID-19 between 2020 and 2023 [version 1; peer review: 1 not approved]. *F1000Research* 2024, **13**:673 ( https://doi.org/10.12688/f1000research.150761.1)

 REBUTTAL

 Firstly the authors would like to thank the reviewer for the time spent providing comments on this paper and the provision of two extremely helpful references. We note there is a significant delay between the receipt of the review and this response. This is partly due to the first two authors being deployed to Rwanda as part of international efforts to support its response to the Marburg outbreak in 2024. We provide our replies below and have edited the paper in line with the recommendations where we can. We have also explained where we can’t make changes largely due to the constraints of using secondary data collected under the Clinical Characterisation Protocol designed by the International Severe Acute Respiratory and Emerging Infections Consortium (ISARIC) and the World Health Organisation (WHO). ^^[1]^^ Our hope is that this revised version addresses the concerns within the limitations of the data and improves the clarity of the findings as well as providing an improved version that may attract a second positive review to enable this paper to be fully published and indexed. 

*PR1. It is unclear to me the extent to which individuals were hospitalised for a specific reason (e.g. due to their cancer ) and identified as having Covid 19 or were hospitalised for respiratory symptoms and identified as having another diagnosis such as cancer. This is particularly important as there are conclusions about different care between these groups and the demographic characteristics of those with cancer are substantially different from others admitted and this may be driven by the reason for admission. Additionally, the implications for the difference in demographic characteristics are not fully explored in the discussion.*

 Response

 Our study uses secondary data collected under the Clinical Characterisation Protocol designed by the International Severe Acute Respiratory and Emerging Infections Consortium (ISARIC) and the World Health Organisation (WHO). This is a strength of our study as it allows analysis of a large, international dataset. However, we also acknowledge the limitations in that, although the target population for the protocol is patients admitted due to COVID-19 illness, the data recorded did not include the primary reason for presentation at the hospital and subsequent admission as a validation of this population. In the Conclusion we acknowledge the lack of certainty of the reason for presenting to hospital and we have edited the paper to highlight this in the Strengths and Limitations section. However, we feel our Results are still relevant, and in line with a number of similar, published studies, as they report the treatment subsequent to admission of patients with confirmed COVID-19 with respect to the presence or absence of cancer. The results of this analysis can help to inform resource planning and non communicable disease management like cancer in future outbreaks of respiratory disease. We do agree that it is unknown if a treatment regime might have differed due to demographics or characteristics at admission and have added this to the Limitations and Discussion of Results.

*PR2. The major results are the comparison of the outcomes of those with cancer versus those without cancer. The authors point out there are other individuals with conditions with increased risk in their comparison group. As such by combining all other groups the approach is likely to underestimate the increase in risk of those with cancer. This is possibly the output in figure 3, however this is not explicitly stated and the reporting of adjusted results for other variables (such as age and sex) appear to be without adjusting for this. In other results this is not addressed, e.g.  Data in Table 2 seems to be a direct comparison between those with and without cancer only.*

 Response

 Thank you for pointing this out. The adjusted hazards ratio for cancer was obtained from a multivariable model that included age, sex, and country income level with no explicit adjustment made for further co-morbidities. We agree data presented in table 2 are crude that are not adjusted for any patient characteristics, however we sought to represent the data in relation to our study question.

 Following your comments we have undertaken two further sensitivity analyses and present the findings below and in the revised paper. First by adjusting for the comorbidity status in addition to age, sex, and income status in the multivariable model to estimate the hazard ratio of cancer on mortality (sensitivity analysis table1). Second, a multivariable model with all the predictors listed in Table 2 included in the analysis (sensitivity analysis table 2 below). The findings from these two sensitivity analyses indicates that the qualitative association between any of the predictors and outcomes remain relatively stable with some or minor differences in the estimated hazards ratio, apart from chronic neurological disorder. However, it has to be cautioned that such a multivariable model with all the predictors included is subject to large missingness (as indicated in sensitivity analysis 2 table) that makes the analysis susceptible to bias due to missing data.

 These results from the two sensitivity analyses are presented as Table 4 and Table 5 in the revised manuscript.

**Sensitivity analysis table 1: **Hazards ratio of mortality among those with cancer, adjusted for comorbidities

 Adjustment set

 Hazards ratio [95% CI]

 Results presented in main manuscript

 Not adjusted for any variables

 (from Table 3)

 1.16 (1.13-1.18)

 Adjusted for age, sex, and income levels

 (from Table 3)

 1.18 (1.15-1.20)

 Sensitivity analyses

 Adjustment set:

 age, sex, income level,

 hypertension, diabetes, COPD, obesity, chronic cardiac diseases, dementia, asthma, neurological disorder, rheumatological disorder

 1.18 (1.14-1.21)

**Sensitivity analysis table 2: **Multivariable model with all the predictors listed in Table 2 included in the analysis (n=102,184 patients, 16,105 events, and 458,363 missing observations excluded from the multivariable analysis).

 Unadjusted hazards ratio

 (from Table 3)

 Adjusted hazards ratio (95% CI)

 Cancer (reference: no)

 1.16 (1.13-1.18)

 1.20 (1.16-1.26)

 60 years and above (reference: 0-59y)

 2.01 (1.98-2.04)

 2.63 (2.50-2.77)

 Diabetes mellitus (ref: no)

 1.4 (1.38-1.42)

 1.20 (1.17-1.24)

 Chronic pulmonary disease (ref: no)

 1.31 (1.28-1.33)

 1.33 (1.28-1.38)

 Male (ref: female)

 1.11 (1.1-1.12)

 1.24 (1.20-1.28)

 Chronic cardiac disease (ref: no)

 1.2 (1.19-1.22)

 1.26 (1.21-1.30)

 Obesity (ref: no)

 0.97 (0.95-0.99)

 1.10 (1.06-1.15)

 Hypertension (ref: no)

 1.37 (1.35-1.38)

 1.10 (1.01-1.14)

 Dementia (ref: no)

 1.51 (1.48-1.55)

 1.16 (1.10-1.22)

 Smoking (ref: no)

 1.04 (1.02-1.06)

 1.04 (1.00-1.08)

 Asthma (ref: no)

 0.93 (0.91-0.95)

 1.03 (0.98-1.08)

 Chronic neurological disorder (ref: no)

 1.13 (1.1-1.16)

 0.95 (0.91-0.99)

 Chronic rheumatological disorder (ref: no)

 1.13 (1.1-1.16)

 0.96 (0.93-1.00)

*PR3. The analysis does not include an examination of change over time of risk associated with cancer. It also does not look at the impact of vaccination on risk (which we become more prevalent over time).  There is literature on changing risk over the course of the pandemic of individuals with cancer - e.g. Ref 1*

*and the impact of vaccination and vaccine response Ref 2 **.***

 Response

 Our study includes patients from January 30, 2020, to January 10, 2023. During this period, COVID-19 underwent significant changes in genomics, treatment, and epidemiology, with vaccines introduced at varying times across countries. However, our dataset lacks genotyping and reliable vaccination information, which are crucial for analyzing temporal changes accurately.

 Without data on these key factors, especially vaccination status, we cannot provide a robust analysis of changes over time. The impact of evolving vaccination rates on outcomes is likely substantial but impossible to calculate with our current data.

 We acknowledge this limitation more explicitly in this revision and our findings have already informed improvements to ISARIC's case report forms for future outbreaks to address these data gaps.

*PR4. It is unclear on the rationale of covariates included in the analysis and why others have been omitted. For example, a key strength is the international nature of this dataset, which allows exploration of differences between countries. It is stated in the paper the authors are unaware of any other analysis looking at outcomes using and international data set. There is between countries differences in provision of health care, even of those with similar income level. However, the rationale for why country income level was chosen and its implications is not discussed or why a more granular variable reflecting country could not be used. Rather it is stated that due to the predominance of cases from high income countries they were unable draw inferences, suggesting that using world bank income classification is not an appropriate variable to use. The authors need to reconsider what is used to assess international differences and include this in the analysis. Other examples, including those mentioned above, vaccine status, other clinical comorbidities, likely variant (or time as a proxy), severity at admission (or length of hospitalization)  etc,*

 Response

 Despite its faults, our original analysis plan included an analysis of differences in patient outcomes between countries with different World Bank classifications as a proxy for the quality of healthcare systems between these two economic realities. Other analyses of this database have used this approach due to the sensitivities and inaccuracies of labelling a select collection of hospitals in participating countries as country-level results. We have added this explanation to the methodology. While we agree that there are huge differences between health care institutions within these national income brackets, we feel that presenting a descriptive analysis is useful to identify a signal of difference that should be explored with a targeted study or datasets that include the details needed. The impact of comorbidities has been explored in our analysis. Unfortunately, we did not have data on vaccination status.

 (There was no PR5 in the peer review report)

*PR6. The presentation of results needs review to ensure it is clear. For example, the Figure 2 Kaplan Meier plot references inclusion of people between 2020-2023 - this is the only reference to a time period, the x axis is labelled as time and is in days. I assume this is the risk from the date of admission, however that is not explicitly stated - as such the presentation is not clear.*

*Note,  If it is the number of days hospitalized, rather than the days since admission, part of the downward trend may be driven by those no longer hospitalized being removed from the analysis as time continues, leaving only those hospitalized for longer being included in survival risk at later timepoints . Without clear description in is challenging to determine if this is a relevant point.*

*Other examples include Figure 3 - while the graphical representation is helpful - it is not possible to ascertain the exact hazard ratio and confidence intervals are omitted*

 Response

 The study population statement clarifies that patients were enrolled between 30 ^th^ January 2020 and 10 ^th^ January 2023 and were followed for up to 30 days from admission.  We agree the labelling and legends of the tables and graphs should be improved to provide more context as standalone figures and tables and have edited the paper accordingly. We have additionally removed Figure 3 as these results are presented with 95% CI in Table 3, and further in Tables 4 and 5.

*PR7. It is stated the presence of cancer was self-reported, It may be that more detail of this process would clarify potential biases, however given findings that the cancer group were, for example, admitted less to ICUs it is conceivable that those presenting with more severe symptoms at the time of recruitment were unable to self-report if they had cancer. Greater clarity on either the process of collecting relevant clinical details or discussion of potential implications of data collection approaches are needed.*

 Response

 Cancer status was obtained from patients who were able to report or family members for many who could not. Those who could not report and did not have family were excluded from the analysis, and hence the potential for bias to arise due to lack of patients’ ability of self-report is likely mitigated in this analysis.

 However, we appreciate that this approach can lead to bias if those with missing self-reported cancer status have different characteristics than those who were included in the analysis. Appropriate analysis requires investigation of the mechanism that led to the missingness of the self-reported cancer status and this was beyond the scope of the current work.

 We have clarified this in the Methods section to make this more explicit and reduce the concern for bias.

 NB we note reference 14 to IDDO is incomplete and the link to the CRF as ref 14 in the Introduction is incorrect it should link to ref 15.

 We have corrected this in our revision. Thank you.

^^[1]^^ https://isaric.org/research/covid-19-clinical-research-resources/covid-19-crf/
